# Not a Deficit, Just Different: Prepulse Inhibition Disruptions in Autism Depend on Startle Stimulus Intensities

**DOI:** 10.1523/ENEURO.0179-24.2024

**Published:** 2024-09-03

**Authors:** Ella Elizabeth Doornaert, Alaa El-Cheikh Mohamad, Gurwinder Johal, Brian Leonard Allman, Dorit Möhrle, Susanne Schmid

**Affiliations:** ^1^Anatomy & Cell Biology, Schulich School of Medicine & Dentistry, University of Western Ontario, London, Ontario N6A 5C1, Canada; ^2^Comparative Biology and Experimental Medicine, University of Calgary, Calgary, Alberta T2N 4Z6, Canada; ^3^Psychology, University of Western Ontario, London, Ontario N6A 5C1, Canada

**Keywords:** animal model, autism, method, sensorimotor gating, sensory filtering, startle

## Abstract

Sensory processing disruptions are a core symptom of autism spectrum disorder (ASD) and other neurological disorders. The acoustic startle response and prepulse inhibition (PPI) are common metrics used to assess disruptions in sensory processing and sensorimotor gating in clinical studies and animal models. However, often there are inconsistent findings on ASD-related PPI deficits across different studies. Here, we used a novel method for assessing changes in startle and PPI in rodents, using the *Cntnap2* knock-out (KO) rat model for neurodevelopmental disorder/ASD that has consistently shown PPI disruptions in past studies. We discovered that not only sex and prepulse intensity but also the intensity of the startle stimulus profoundly impacts whether PPI deficits are evident in the *Cntnap2* KO rat or not. We show that rats do not universally exhibit a PPI deficit; instead, impaired PPI is contingent on specific testing conditions. Notably, at lower startle stimulus intensities, *Cntnap2* KO rats not only demonstrated intact PPI but also exhibited evidence of enhanced PPI compared with their wild-type counterparts. This finding emphasizes the importance of considering specific testing conditions when evaluating startle and PPI in the context of ASD and other neuropsychiatric conditions and might explain some of the inconsistencies between different studies.

## Graphical abstract

**Figure:**
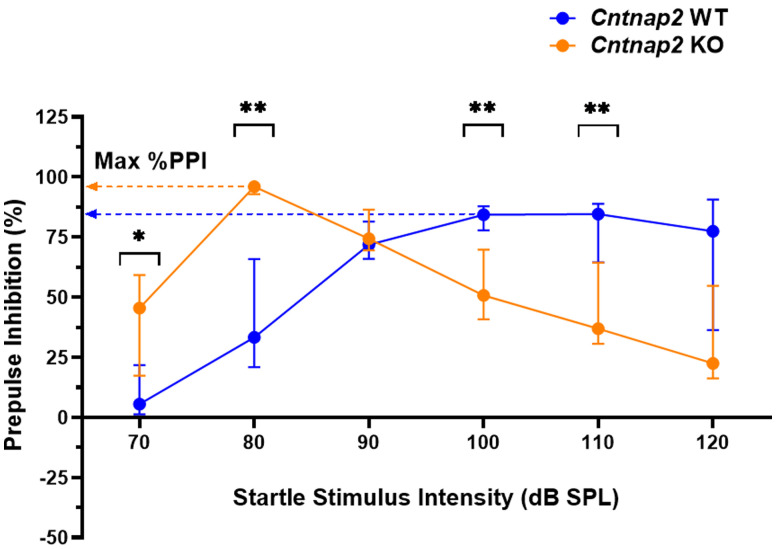
Visual Abstract

## Significance Statement

The present study extends traditional approaches to evaluating sensory processing using startle and prepulse inhibition (PPI) by showing that startle and PPI disruptions are contingent upon specific testing parameters. Compared with conventional PPI testing where only prepulse levels and interstimulus intervals might vary, we here show that animals consistently reported to have PPI deficits do not have a general sensorimotor gating deficit but intact, and potentially even enhanced, PPI at lower startle intensities. This has a widespread impact on PPI testing and the interpretation of PPI results, given the broad use in animal models of various neurodevelopmental conditions, alongside the translational relevance to clinical settings.

## Introduction

**Figure 1.**
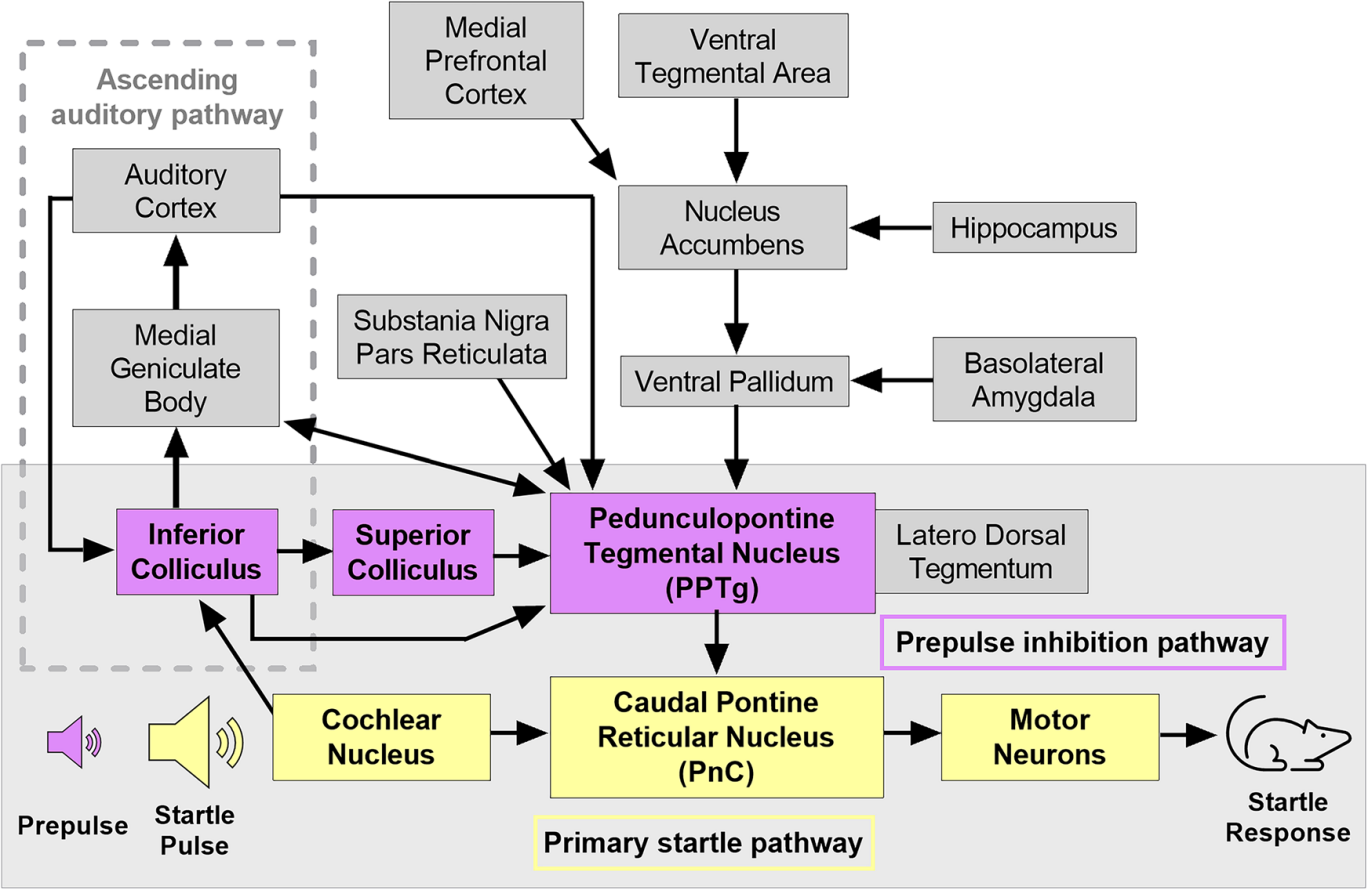
Hypothetical model of the acoustic startle response and PPI. The primary acoustic startle pathway (yellow) consists of the cochlear nucleus, caudal pontine reticular nucleus (PnC), and spinal cord motor neurons. The primary circuit responsible for PPI (purple) involves the cochlear nucleus and inferior colliculus of the ascending auditory pathway (gray dotted frame) and pedunculopontine tegmental nucleus (PPTg). PPTg inhibitory projections to the PnC are assumed to mediate the attenuation of the startle response. Additional modulatory input to the PPTg includes projections from the medial geniculate body, auditory cortex, substania nigra pars reticulata, and ventral pallidum. Figure modified from Möhrle et al. (2021) and El-Cheikh Mohamad et al. (2023). Based on data and information from Koch (1999), Fulcher et al. (2020), Gómez-Nieto et al. (2020), and Weible et al. (2020).

Disruptions in sensory filtering and sensorimotor gating are core symptoms in several neuropsychiatric disorders and neurological diseases. Autism spectrum disorder (ASD) is a neurodevelopmental condition characterized by deficits in social communication and interaction, along with restricted and repetitive patterns of behavior or interests (American Psychiatric Association, 2013) that go along with sensory processing disruptions (Leekam et al., 2007). Sensory filtering and sensorimotor gating are commonly assessed by measuring the acoustic startle response and prepulse inhibition of startle (PPI) in affected humans and in respective animal models (Kohl et al., 2014; Sinclair et al., 2017; Takahashi et al., 2017; Möhrle et al., 2020). Startle responses involve muscle contractions in response to a sudden loud noise and are mediated by a well-defined brainstem circuit consisting of the cochlear root nucleus, pontine reticular nucleus (PnC), and spinal cord motor neurons (Fig. 1; for review, see Koch, 1999; Zheng and Schmid, 2023). The startle-mediating giant neurons of the PnC serve as the sensorimotor interface in which cochlear root neurons synapse with premotor neurons (Koch et al., 1992; Lingenhöhl and Friauf, 1994). PPI refers to the reduction in the startle response by a brief (nonstartling) stimulus (for review, see Gómez-Nieto et al., 2020). PPI is assumed to be mediated by a feedforward inhibitory neural circuit that begins with cochlear neurons signaling to the inferior colliculus, which then project to the pedunculopontine tegmental nucleus (PPTg; Koch, 1999). PPI is presumably mediated through inhibitory projections from the PPTg to the PnC (Koch, 1999). Given the highly conserved nature of these pathways, utilizing startle and PPI testing in rodent models of ASD can provide valuable insights into the neural mechanisms underlying changes in sensory processing and filtering observed in autistic humans.

**Figure 2.**
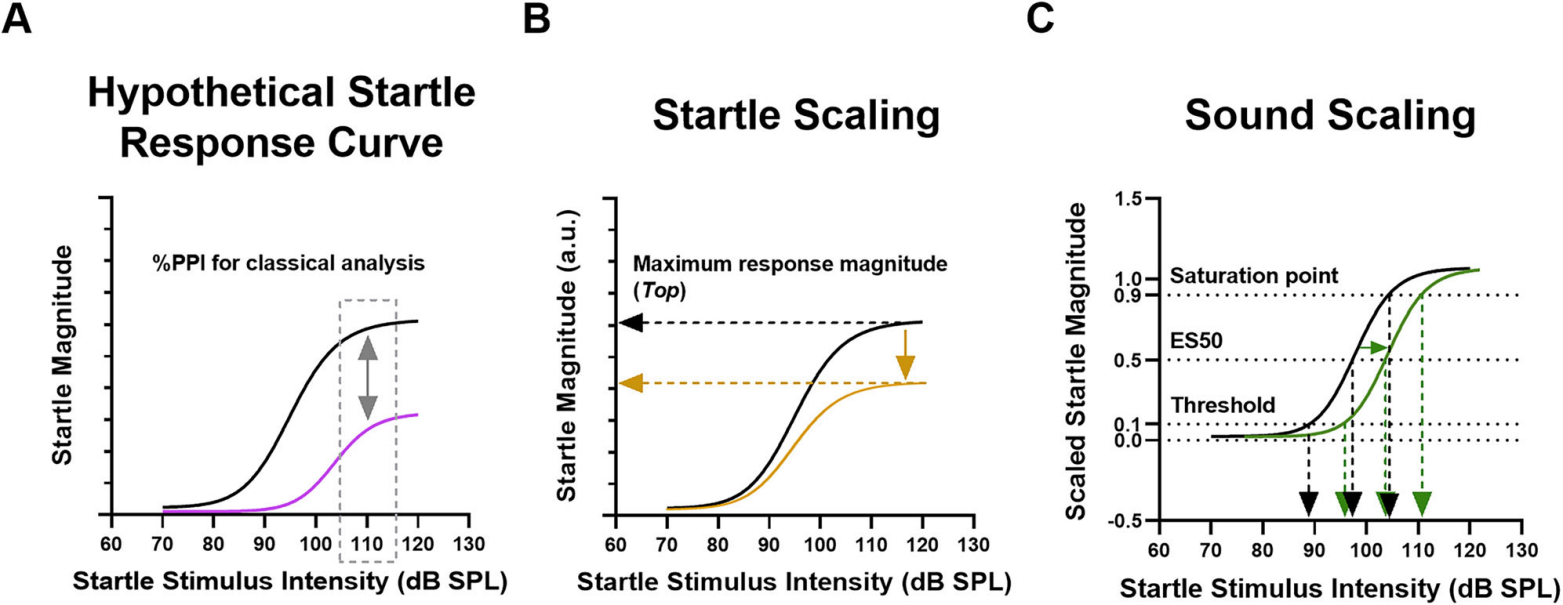
Hypothetical model of how the baseline startle response curve is changed by startle and sound scaling. ***A***, Hypothetical startle response curve (black) is scaled by a prepulse resulting in a downward and rightward shift (purple), indicative of startle and sound scaling, respectively. Classical analyses of PPI calculate the %PPI using one startle stimulus intensity (gray dotted frame) and consequently, cannot be informative of scaling components. ***B***, Startle scaling results from a reduction in response amplitude and is shown by a downward shift in the startle response curve (orange). Startle scaling is determined by the change in the maximum response magnitude (Top). ***C***, Sound scaling results from a reduction in sound sensitivity and is shown by a rightward shift in the startle response curve (green). Sound scaling is determined by the change in threshold, ES50, and saturation point. Figure modified from El-Cheikh Mohamad et al. (2023). Based on data and information from Martin-Iverson and Stevenson (2005), Miller et al. (2021), and El-Cheikh Mohamad et al. (2023).

One ASD animal model that has been consistently shown to have increased startle and reduced PPI of startle is the *Cntnap2* knock-out rat (Scott et al., 2018, 2020, 2022; Möhrle et al., 2021; El-Cheikh Mohamad et al., 2023; Haddad et al., 2023). Homozygous loss-of-function mutation in the *CNTNAP2* gene has been linked to a syndromic form of ASD, and multiple studies have identified other *CNTNAP2* mutations as risk factors for ASD (Strauss et al., 2006; Poot, 2017). In the present study, we used a novel comprehensive method to assess startle and PPI in more detail in the *Cntnap2* KO rat (Miller et al., 2021; El-Cheikh Mohamad et al., 2023). This method uses multiple startle stimulus intensities for PPI assessment, incorporates more trials per animal, and treats startle responses as nonparametric data. Most importantly, it considers the baseline startle response curve as a sigmoidal input/output (I/O) function in which the startle stimulus intensity (input) results in the corresponding response magnitude (output; Fig. 2*A*; Martin-Iverson and Stevenson, 2005; Miller et al., 2021). In the presence of a prepulse, the entire baseline I/O curve is scaled by two different components: startle and sound scaling. Startle scaling, as shown by a downward shift in the I/O curve, results from a reduction in response amplitude (Fig. 2*B*). Sound scaling, as shown by a rightward shift in the I/O curve, results from a reduction in sound sensitivity (Fig. 2*C*). Startle and sound scaling are thought to relate to the sensory processing and motor output mechanisms of PPI, respectively (Miller et al., 2021). Impaired startle scaling suggests dysfunction in the primary startle pathway, presumably in the PnC, while impaired sound scaling likely pertains to regions involved in PPI, including those upstream to the PnC (Koch et al., 1992; Lee et al., 1996; Gómez-Nieto et al., 2020).

Using this extensive method, both scaling components have previously been investigated in the *Cntnap2* KO rat, and it was found that while startle scaling is intact, sound scaling is disrupted in *Cntnap2* KO rats, especially at higher startle sound intensities and not so evidently at lower startle sounds (El-Cheikh Mohamad et al., 2023). To further investigate this, we used an extended range of startle stimulus intensities in this study to analyze sound scaling distributions in more detail. Furthermore, since previous studies of Miller et al. (2021) found unexpected novel sex effects on PPI in a fragile X rat model using this scaling analysis, we introduced new statistical tools, ARTool and ART-C, enabling an in-depth examination of sex effects (Wobbrock et al., 2011; Elkin et al., 2021). Through this, we discovered that sex, prepulse intensity, and startle stimulus intensity profoundly impact whether PPI deficits are evident or not. Our results highlight that the *Cntnap2* KO rat does not generally exhibit a sensorimotor gating deficit, but rather that impaired PPI is contingent on specific startle sound intensities, which could also explain the notoriously inconsistent findings on PPI deficits across animal models and different labs.

## Materials and Methods

### Animals

Adult (postnatal day 75+) male (*M*) and female (*F*) Sprague Dawley wild-type rats (*Cntnap2* WT; *M* = 11; *F* = 10) and homozygous *Cntnap2* KO rats (*M* = 21; *F* = 19) were used. *Cntnap2* KO rats were bred from heterozygous *Cntnap2* KO breeders (*M* = 10; *F* = 9) or homozygous *Cntnap2* KO breeders (*M* = 11; *F* = 10). Initial heterozygous *Cntnap2* KO breeders were obtained from Horizon Discovery. Rats were housed in open-top cages in groups of two to three with continuous access to food and water. Holding rooms were temperature controlled and followed a 12 h light/dark cycle. Behavioral assessments were conducted during the light phase of this cycle (from 7:00 to 19:00 h). All procedures were approved by the University of Western Ontario Animal Care Committee and were under the guidelines established by the Canadian Council on Animal Care.

### Acoustic startle responses

The acoustic startle response and PPI were assessed using the Med Associates startle boxes and accompanying system. Startle and PPI protocols were constructed as previously reported (Miller et al., 2021; El-Cheikh Mohamad et al., 2023). In brief, animals were placed in plexiglass tubes fixated on pressure-sensitive platforms. Before testing, animals underwent three sessions consisting of 3 min of handling by the experimenter followed by a 5 min acclimation session in the startle box with only background noise (65 dB sound pressure level, SPL, white noise). After acclimation, a session was conducted to determine the I/O function to inform adjustments in the gain of the startle platform to achieve optimal readings. The I/O function consisted of 12 startle stimuli presented to the animals, ranging from 65 to 120 dB (20 ms white noise; 5 dB increments), plus two stimuli used in the PPI protocol (75 and 85 dB, 4 ms white noise); stimuli were presented in pseudorandomized order.

After determining the I/O function, animals were tested twice daily for 5 consecutive days. Each testing session comprised three distinct blocks: acclimation, habituation, and PPI. The first block was a 5 min acclimation period with only background noise (65 dB white noise). The second block habituated the animals to the startle stimuli by presenting animals with 12 trials of a 110 dB startle stimulus (20 ms white noise) with a variable intertrial interval of 10–15 s. In the third block, PPI was examined using trials pairing a nonstartling prepulse (75 or 85 dB, 4 ms white noise) and a startle stimulus (70, 80, 90, 100, 110, or 120 dB, 20 ms white noise) at a fixed interstimulus interval of 100 ms. In addition, a startle-alone stimulus was presented. These trials, arranged in a pseudorandomized order, had variable intertrial intervals of 10–15 s and were repeated four times each per testing session, resulting in 84 trials per testing session and 40 repetitions per trial type across all 10 testing sessions.

### Data analysis

The startle magnitude was defined as the maximum peak-to-peak value. Before statistical analyses, absolute startle magnitudes were calculated for each rat correcting for the gain factor. Subsequent analysis was based on Martin-Iverson and Stevenson (2005) and Miller et al. (2021). Startle reactivity was assessed over the range of startle stimuli by fitting each animal's responses to a sigmoidal regression function in GraphPad Prism 9.3.1 (Nonlinear regression; Method: Sigmoidal, 4PL, *X* is concentration; Method: Least squares regression; Initial values: choose automatically; Confidence: Unstable parameters and ambiguous fits as Neither option; Diagnostics: default values including Adjusted *R* Squared, RMSE, and tests of normality; see also Möhrle et al. 2021) with the following equation:

$$Y=\mathrm{Bottom}+X^{\mathrm{Hillslope}}\left(\frac{\mathrm{Top}-\mathrm{Bottom}}{X^{\mathrm{Hillslope}}+\;\mathrm{ES}50^{\mathrm{Hillslope}}}\right),$$

where *Y* is the startle response magnitude, Top is the maximum startle response magnitude, and Bottom is the minimum response magnitude. *X* is the startle stimulus intensity (dB SPL) required to produce a certain *Y* value (in arbitrary units). ES50 is the sound intensity (dB SPL) required to maintain the half-maximum response. Hillslope is the slope of the curve. Parameters of interest were derived from the equation to evaluate and compare differences in baseline startle and PPI. This includes the maximum startle response (Top), startle threshold (10% of maximum threshold), ES50, and saturation point (90% of maximum startle). In this sigmoidal regression analysis, GraphPad Prism provided the standard error of regression using Sy.x, which serves as an estimate of the goodness-of-fit for models involving two or more parameters.

Startle scaling was determined by changes to the maximum startle response, or Top (El-Cheikh Mohamad et al., 2023). Each animal's responses were fitted to a sigmoidal regression function, following the previously described procedure (including Constrain: Bottom is constant equal to 0).

Sound scaling was determined by changes to the threshold, ES50, and saturation point (Martin-Iverson and Stevenson, 2005; Möhrle et al., 2021; El-Cheikh Mohamad et al., 2023). Startle responses for each animal and prepulse condition were scaled between 0 and 1. To scale the startle magnitudes at each startle stimulus intensity (*X*), we used the following equation: (startle magnitude at *X* − startle magnitude at 70 dB startle stimulus) / (startle magnitude at 120 dB startle stimulus − startle magnitude at 70 dB startle stimulus).

Scaled values were then fitted to the sigmoidal regression function using the same procedure as above (except, Constrain: Bottom is constant equal to 0 and Top is constant equal to 1). ES50 was provided by the regression. The threshold and saturation point were calculated in MATLAB R2022a by rearranging Equation 2 to solve for *X*. The threshold *Y* value was set to 10% of the Top, and the saturation point *Y* value was set to 90% of the Top.

$$X=\sqrt[{\mathrm{Hillslope}}]{\frac{\left(Y-\mathrm{Bottom})\times (\mathrm{ES}50^{\mathrm{Hillslope}}\right)}{\mathrm{Top}-Y}}.$$

The slope (Hillslope) of the normalized startle curves can be used as a metric of reflex efficiency (Martin-Iverson and Stevenson, 2005). The slope was also provided by the regression.

For comparison with traditional PPI analysis, the percent PPI was calculated using the startle magnitudes obtained during the PPI block:

$$\text{\%}\mathrm{PPI}=\;\left(1-\;\frac{\mathrm{startle}\;\mathrm{magnitude}\;\mathrm{with}\;\mathrm{prepulse}}{\mathrm{baseline}\;\mathrm{startle}\;\mathrm{magnitude}}\right)\times 100\text{\%}.$$

### Statistical analysis

Data are presented as group medians with errors indicating interquartile range (IQR). Outlier analysis was performed in IBM SPSS (version 26) with the PPI startle and sound scaling parameters for each genotype and sex. Through boxplot assessment, extreme outliers were identified as those exceeding 3 IQR from the median and were subsequently excluded from all analyses; four *Cntnap2* WT rats (*M* = 4; *F* = 0) and six *Cntnap2* KO rats (*M* = 4; *F* = 2) were found to be extreme outliers and removed. Subsequent statistical analyses were performed in GraphPad Prism 9.3.1 and RStudio 2022.07.2, and figures were generated in GraphPad Prism 9.3.1. To determine the main effects and interactions, we employed ARTool (Aligned Rank Transform, ART) to align-and-rank data for nonparametric factorial ANOVA, and ART-C for post hoc pairwise comparisons (Wobbrock et al., 2011; Elkin et al., 2021). Statistical tests following the ART were based on the experimental design and included univariant analysis of variance (two-way ANOVA or repeated-measures ANOVA, as appropriate). Post hoc comparisons were conducted using Sidak's multiple-comparisons test when appropriate. For measures in which there was no effect of sex or an interaction effect involving sex, sex was collapsed to examine the effect of genotype. These measures were assessed using a Mann–Whitney test. The chosen statistical significance level was *α* = 0.05. Resulting *p* values are reported in the figure captions using no asterisk or ns for nonsignificance: *p < 0.05; **p < 0.01; ***p < 0.0001.

## Results

### *Cntnap2* KO rats have increased startle reactivity that is associated with a leftward shift in males but not females

**Figure 3.**
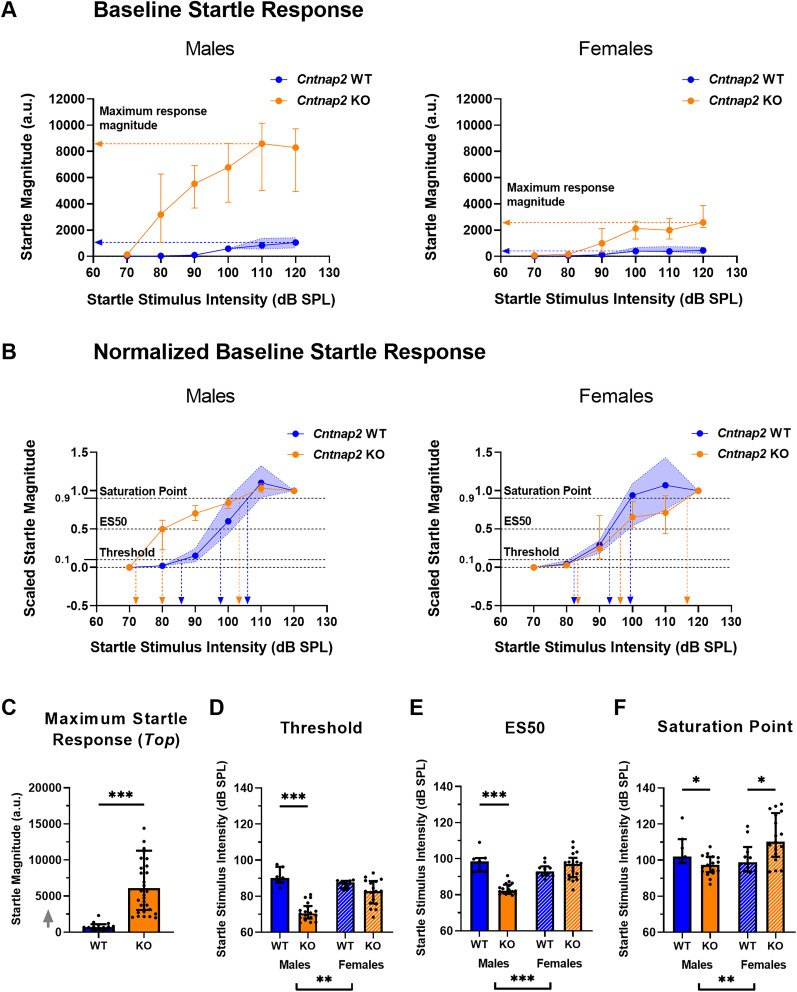
*Cntnap2* KO rats have increased startle reactivity that is associated with a leftward shift of the startle I/O curve in males but not females. *Cntnap2* WT rats are represented in blue and *Cntnap2* KO rats in orange. All graphs show group medians from the 40 repetitions per startle stimulus SPL with error bars representing IQR. Scatterplots show individual values. ***A***, Baseline startle response curves. The colored arrows point to the maximum startle response value (Top) for each genotype. *Cntnap2* WT IQR is visualized as the blue-shaded area. Goodness of fit Sy.x: male *Cntnap2* WT = 449.7, male *Cntnap2* KO = 2,336, female Cntnap2 WT = 181.1, female *Cntnap2* KO = 881.9. ***B***, Scaled startle response curve. The colored arrows point to the threshold, ES50, and saturation point for each genotype. *Cntnap2* WT IQR is visualized as the blue-shaded area. Goodness of fit Sy.x: male *Cntnap2* WT = 0.2048, male *Cntnap2* KO = 0.1257, female *Cntnap2* WT = 0.1956, female *Cntnap2* KO = 0.1937. ***C***, Maximum startle response (Top). The gray arrow indicates that there are values outside the limits of the *y*-axis, but graphs were zoomed in to visualize the data more clearly. Sex was collapsed to examine the effect of genotype. *Cntnap2* KO rats showed a greater maximum startle response magnitude than *Cntnap2* WT rats. ***D***, Threshold. Male but not female *Cntnap2* KO rats showed a lower response threshold than *Cntnap2* WT rats. ***E***, ES50. Male but not female *Cntnap2* KO rats showed a lower ES50 than *Cntnap2* WT rats. ***F***, Saturation point. *Cntnap2* KO males showed a lower saturation point than *Cntnap2* WT males. *Cntnap2* KO females showed a higher saturation point than *Cntnap2* WT females. *p < 0.05, **p < 0.01, ***p < 0.0001, no asterisk or ns indicates nonsignificance of the comparison.

The effect of *Cntnap2* knock-out on baseline startle magnitude was assessed using the extracted parameters of the baseline startle I/O curves: maximum startle response (Top; Fig. 3*A*), threshold, ES50, and saturation point (Fig. 3*B*). There was no effect of sex or interaction effect between genotype and sex on the maximum startle response (sex, *p *= 0.1320, *F*_(1,47)_ = 2.350; genotype × sex, *p *= 0.1262, *F*_(1,47)_ = 2.424). Therefore, sex was collapsed to examine the effect of genotype on startle magnitude. *Cntnap2* KO rats had a greater maximum startle response than their WT counterparts (*p *< 0.0001; Fig. 3*A*,*C*).

For the startle threshold, there was a significant main effect of genotype and sex, as well as an interaction effect between genotype and sex (genotype, *p *< 0.0001, *F*_(1,47)_ = 62.35; sex, *p *= 0.0001, *F*_(1,47)_ = 17.88; genotype × sex, *p *= 0.0001, *F*_(1,47)_ = 18.80). Whereas *Cntnap2* KO males showed a lower startle threshold than WT males, there was no difference between *Cntnap2* WT and KO females (males, *p *< 0.0001; females, *p *= 0.1283; Fig. 3*B*,*D*). Similarly, for the ES50 there was a significant effect of genotype and sex, as well as an interaction effect between genotype and sex (genotype, *p *= 0.0003, *F*_(1,47)_ = 15.69; sex, *p *< 0.0001, *F*_(1,47)_ = 26.91; genotype × sex, *p *< 0.0001, *F*_(1,47)_ = 30.12). Like threshold, *Cntnap2* KO males exhibited a lower ES50 than WT males, but the ES50 did not differ between female *Cntnap2* WT and KO rats (males, *p *< 0.0001; females, *p *= 0.3376; Fig. 3*B*,*E*). Finally, there was also a significant effect of sex and an interaction between genotype and sex on the saturation point (sex, *p* = 0.0077, *F*_(1,47)_ = 7.758; genotype × sex, *p* = 0.0018, *F*_(1,47)_ = 10.92). Whereas *Cntnap2* KO males showed a lower saturation point than WT males, *Cntnap2* KO females had a higher saturation point than WT females (males, *p *= 0.0438; females, *p *= 0.0100; Fig. 3*B*,*F*).

There was an effect of genotype on the slope, but no effect of sex or interaction effect between genotype and sex (genotype, *p *= 0.0001, *F*_(1,47)_ = 19.47; sex, *p *= 0.1515, *F*_(1,47)_ = 2.125; genotype × sex, *p *= 0.3513, *F*_(1,47)_ = 0.8863). Consequently, sex was collapsed to examine the effect of genotype. *Cntnap2* KO rats had a lower slope than WT rats (*p *< 0.0001; data not shown), indicating a lower reflex efficiency.

In summary, we found a heightened startle response in both male and female *Cntnap2* KO rats (startle scaling), as indicated by an increased maximum response (Top) of the startle I/O function. Moreover, the startle response I/O curves of *Cntnap2* KO rats reveal a leftward shift in males (sound scaling), as indicated by the lower startle stimulus intensities for threshold, ES50, and saturation. In contrast, in female *Cntnap2* KO rats, we did not observe this left shift of the startle I/O function but rather a right shift of the saturation point only.

### Impaired prepulse inhibition in the *Cntnap2* KO rat

**Figure 4.**
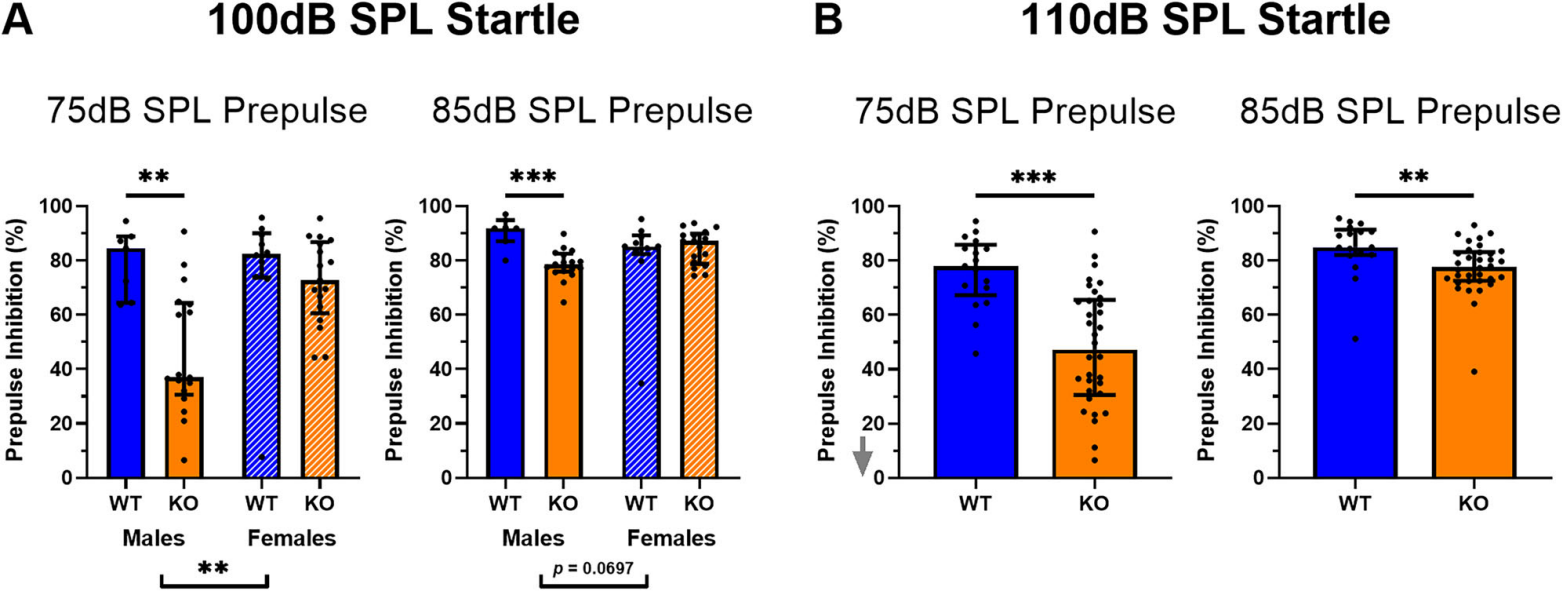
Classical analysis confirms PPI deficits in *Cntnap2* KO rats. *Cntnap2* WT rats are represented in blue and *Cntnap2* KO rats in orange. Scatterplots represent individual values and black lines represent the median with error bars as IQR. The gray arrows indicate that there are values outside the limits of the *y*-axis, but graphs were zoomed in to visualize the data more clearly. ***A***, Classical %PPI analysis at 100 dB startle stimulus. Male but not female *Cntnap2* KO rats showed a PPI deficit with the 75 and 85 dB prepulse. ***B***, Classical %PPI analysis at 110 dB startle stimulus. Sex was collapsed to examine the effect of genotype. *Cntnap2* KO rats showed a PPI deficit with a 75 and 85 dB prepulse. *p < 0.05, **p < 0.01, ***p < 0.0001, no asterisk or ns indicates nonsignificance of the comparison.

First, classical analysis of PPI was performed with a 100 and 110 dB startle stimulus with a 75 and 85 dB prepulse (Fig. 4*A*,*B*). This was done by applying the %PPI formula to the median response of the 40 repetitions obtained for these conditions throughout the PPI sessions. At the 100 dB startle stimulus with a 75 dB, there were main effects of genotype and sex, as well as an interaction effect between genotype and sex on the %PPI (genotype, *p *= 0.0005, *F*_(1,47)_ = 14.06; sex, *p *= 0.0056, *F*_(1,47)_ = 8.438; genotype × sex, *p *= 0.0164, *F*_(1,47)_ = 6.201). At the 100 dB startle stimulus with an 85 dB, there was a main effect of genotype and an interaction effect between genotype and sex on the %PPI (genotype, *p *= 0.0048, *F*_(1,47)_ = 8.757; genotype × sex, *p *= 0.0037, *F*_(1,47)_ = 9.308). With both the 75 and 85 dB prepulse at the 100 dB startle, *Cntnap2* KO males showed a lower %PPI than WT males, but there was no difference between *Cntnap2* WT and KO females (males 75 dB prepulse, *p *= 0.0010; females 75 dB prepulse, *p *= 0.4753; males 85 dB prepulse, *p *< 0.001; females 85 dB prepulse, *p *> 0.9999; Fig. 4*A*).

With the 110 dB startle stimulus, there was a main effect of genotype on the %PPI for both the 75 and 85 dB prepulse (75 dB prepulse, *p *< 0.0001, *F*_(1,47)_ = 24.59; 85 dB prepulse, *p *= 0.0025, *F*_(1,47)_ = 10.19). Since there was no effect of sex or interaction effect of genotype and sex on the %PPI, sex was collapsed for these conditions (75 dB prepulse sex, *p *= 0.7441, *F*_(1,47)_ = 0.1078; 75 dB prepulse genotype × sex, *p *= 0.4071, *F*_(1,47)_ = 0.6997; 85 dB prepulse sex, *p *= 0.9343, *F*_(1,47)_ = 0.0069; 85 dB prepulse genotype × sex, *p *= 0.7940, *F*_(1,47)_ = 0.0689). At the 110 dB startle stimulus with both a 75 and 85 dB prepulse, *Cntnap2* KO rats showed a lower %PPI than WT rats (75 dB prepulse, *p *< 0.0001; 85 dB prepulse, *p *= 0.0012; Fig. 4*B*). Therefore, using classical %PPI analysis, *Cntnap2* KO rats exhibited PPI deficits dependent on sex and startle stimulus intensity. At the 100 dB startle stimulus, male but not female *Cntnap2* KO rats showed a PPI deficit with both a 75 and 85 dB prepulse. At the 110 dB startle stimulus, both male and female *Cntnap2* KO rats showed a PPI deficit with both prepulse intensities.

### Intact startle scaling by prepulses in the *Cntnap2* KO rat

**Figure 5.**
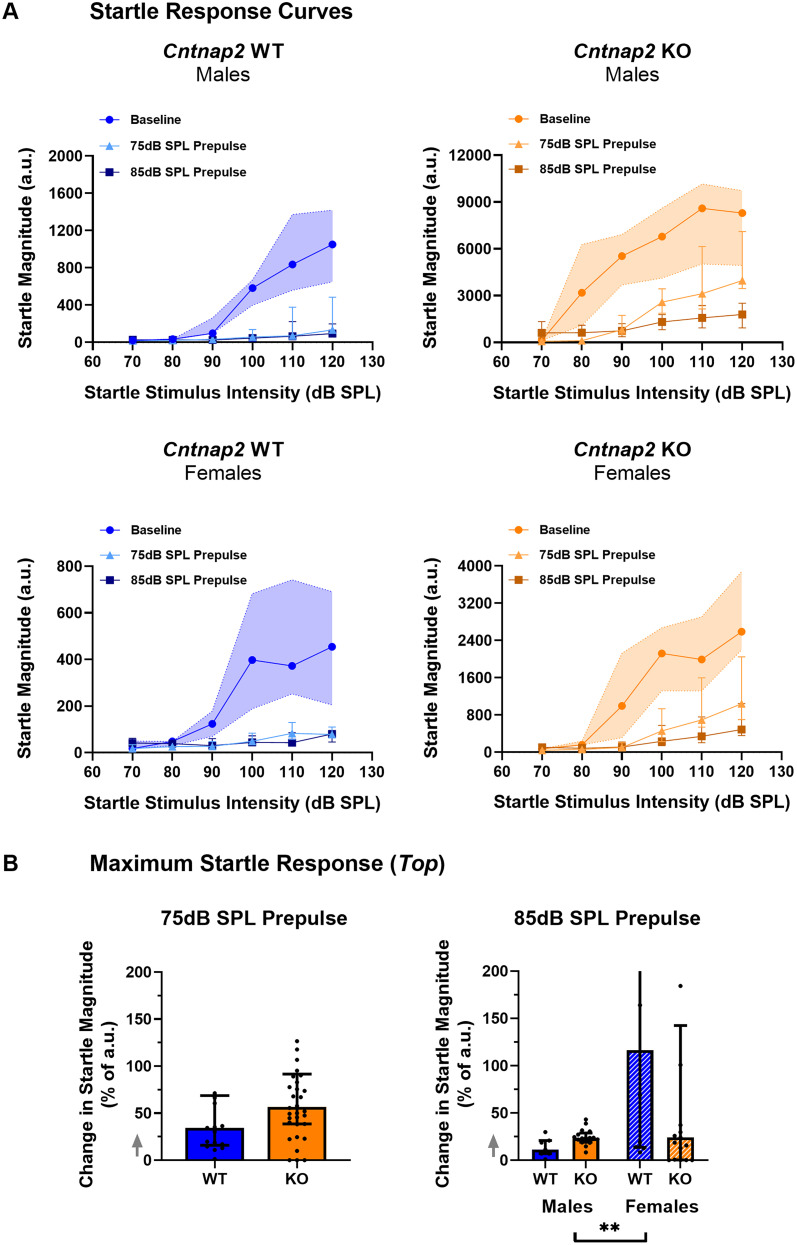
*Cntnap2* KO rats show intact startle scaling through prepulses. *Cntnap2* WT rats are represented in blue and *Cntnap2* KO rats in orange. All graphs show group medians from the 40 repetitions per startle stimulus SPL with error bars representing IQR. Scatterplots show individual values. ***A***, Startle response curves for baseline and prepulse conditions (75 and 85 dB) for *Cntnap2* WT and KO rats. Note: *Y*-axis scales for *Cntnap2* WT and KO rats are different as *Cntnap2* KO rats have a greater baseline startle response magnitude than WT rats. Baseline IQR is visualized as the shaded area. Goodness of fit Sy.x: Cntnap2 WT males baseline = 449.7, 75 dB = 249.6, 85 dB = 54.27, Cntnap2 KO males baseline = 2,336, 75 dB = 1,739, 85 dB = 641.1, *Cntnap2* WT females baseline = 181.1, 75 dB = 44.16, 85 dB = 27.03, *Cntnap2* KO females baseline = 881.9, 75 dB = 513.5, 85 dB = 242.9. ***B***, Maximum startle response (Top). The gray arrows indicate that there are values outside the limits of the *y*-axis, but graphs were zoomed in to visualize the data more clearly. For the 75 dB prepulse, data was collapsed across sex to examine the effect of genotype. The change in the maximum startle response (Top) due to a prepulse did not differ between *Cntnap2* KO and WT rats. *p < 0.05, **p < 0.01, ***p < 0.0001, no asterisk or ns indicates nonsignificance of the comparison.

To examine whether the deficit in PPI is caused by impaired startle or impaired sound scaling, we first assessed startle scaling as the change in the maximum startle response (Top) between the baseline startle and prepulse response curves (Fig. 5*A*). To do this, we calculated the Top value of the startle response I/O curve with a prepulse as a percent of the Top value of the baseline startle response I/O curve for both the 75 and 85 dB prepulse condition. With a 75 dB prepulse, there was no effect of sex or interaction effect between genotype and sex on the change in maximum startle response (sex, *p *= 0.7287, *F*_(1,47)_ = 0.1218; genotype × sex, *p *= 0.6808, *F*_(1,47)_ = 0.1713). Therefore, data was collapsed across sex to examine the effect of genotype. The change in the maximum startle response when a 75 dB prepulse was preceding the startle stimulus was not different between *Cntnap2* WT rats and KO rats (*p *= 0.1039; Fig. 5*B*). With an 85 dB prepulse, there was a significant effect of sex on the change in the maximum startle response, so sex was assessed for post hoc analysis (sex, *p *= 0.0066, *F*_(1,47)_ = 8.089). As with a 75 dB prepulse, the change in maximum startle response through an 85 dB prepulse preceding the startle pulse was not different between *Cntnap2* WT and KO rats, whether male or female (males, *p *= 0.2474; females, *p *= 0.9937). In summary, *Cntnap2* KO rats exhibited intact startle scaling through a prepulse as indicated by a similar change from baseline in Top values as WT rats.

### Sound scaling by prepulses differs between *Cntnap2* KO and WT rats

**Figure 6.**
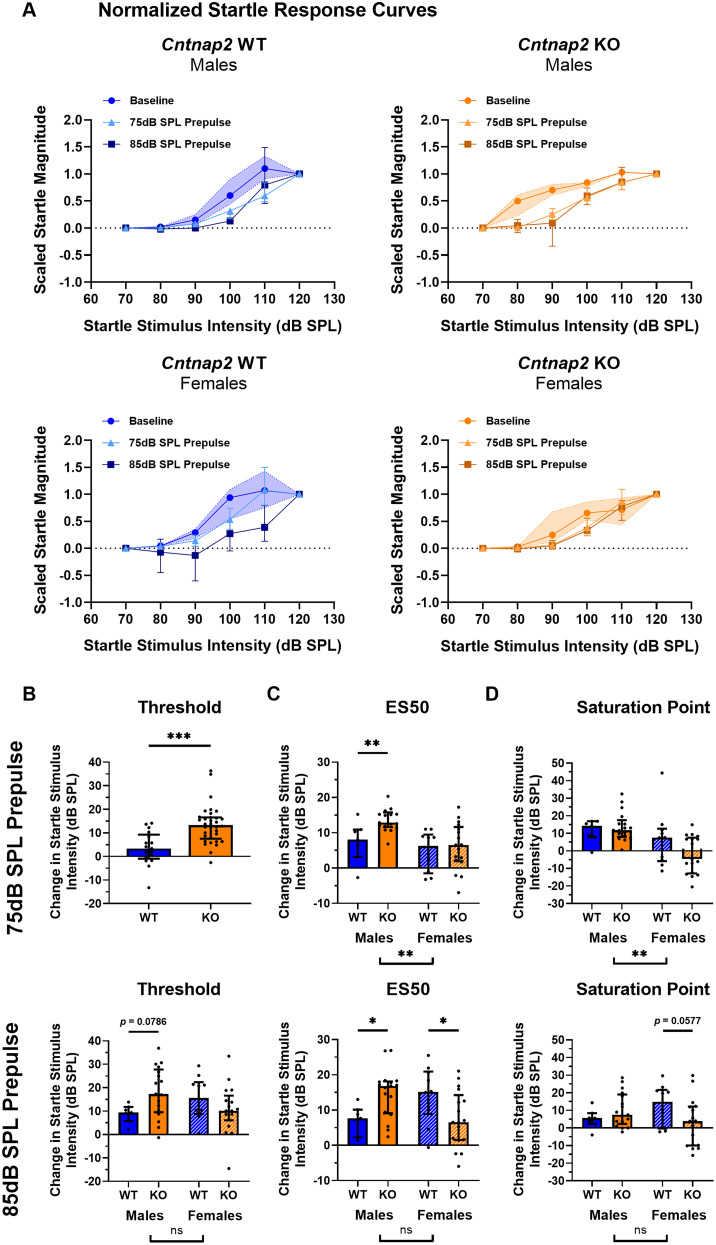
*Cntnap2* KO rats have greater sound scaling by a prepulse at lower startle stimulus intensities and mildly impaired sound scaling at higher startle stimulus intensities. *Cntnap2* WT rats are represented in blue and *Cntnap2* KO rats in orange. All graphs show group medians from the 40 repetitions per startle stimulus SPL with error bars representing IQR. Scatterplots show individual values. ***A***, Scaled startle response curves for baseline and prepulse conditions (75 and 85 dB) for *Cntnap2* WT and KO rats. Baseline IQR is visualized as the shaded area. Goodness of fit Sy.x: *Cntnap2* WT males baseline = 0.2048, 75 dB = 0.09425, 85 dB = 0.2019, *Cntnap2* KO males baseline = 0.1257, 75 dB = 0.1458, 85 dB = 0.3584, *Cntnap2* WT females baseline = 0.1956, 75 dB = 0.2305, 85 dB = 0.9432, *Cntnap2* KO females baseline = 0.1937, 75 dB = 0.1971, 85 dB = 0.1531. ***B***, Change in threshold with a prepulse from baseline. For the 75 dB prepulse, sex was collapsed to examine the effect of genotype. With a 75 dB prepulse, *Cntnap2* KO rats showed a greater change in threshold than WT rats. With an 85 dB prepulse, the change in threshold did not differ between *Cntnap2* KO and WT females. However, the change in threshold for *Cntnap2* KO males was trending toward being significantly greater than WT males. ***C***, Change in ES50 with a prepulse from baseline. With a 75 and 85 dB prepulse, Cntnap2 KO males showed a greater change in ES50 than WT males. *Cntnap2* KO and WT females did not differ in the change in ES50 with a 75 dB prepulse, but with an 85 dB prepulse, *Cntnap2* KO females had a reduced change in ES50 than WT females. ***D***, Change in saturation with a prepulse from baseline. The change in saturation did not differ between *Cntnap2* KO and WT males with a 75 or 85 dB prepulse or between *Cntnap2* KO and WT females with a 75 dB prepulse. However, with an 85 dB prepulse, the change in saturation for *Cntnap2* KO females was trending toward being significantly lower than WT females. *p < 0.05, **p < 0.01, ***p < 0.0001, no asterisk or ns indicates nonsignificance of the comparison.

To examine sound scaling, we assessed the change in threshold, ES50, and saturation point of the scaled startle response curves resulting in using a 75 and 85 dB prepulse from baseline (Fig. 6*A*). With a 75 dB prepulse, there was an effect of genotype, but no effect of sex or an interaction effect between genotype and sex on the change in threshold (genotype, *p *< 0.0001, *F*_(1,47)_ = 21.24; sex, *p *= 0.2659, *F*_(1,47)_ = 1.268; genotype × sex, *p *= 0.4412, *F*_(1,47)_ = 0.6033). Therefore, data was collapsed across sex to examine the effect of genotype. Interestingly, *Cntnap2* KO rats showed a greater change in threshold than WT rats (*p *< 0.0001; Fig. 6*B*), indicating stronger sound scaling. With an 85 dB prepulse, there was an interaction effect between genotype and sex on the change in threshold (genotype × sex, *p *= 0.0141, *F*_(1,47)_ = 6.495). Here, *Cntnap2* KO females did not differ from WT females in terms of changes in threshold; however, *Cntnap2* KO males were trending toward having a greater change in threshold than WT males (males, *p *= 0.0786; females, *p *= 0.6149).

Regarding the change in ES50, there were significant main effects of genotype and sex with a 75 dB prepulse, as well as interaction between genotype and sex on the change in ES50 with an 85 dB prepulse (75 dB prepulse genotype, *p *= 0.0307, *F*_(1,47)_ = 4.962; 75 dB prepulse sex, *p *= 0.0003, *F*_(1,47)_ = 15.20; 85 dB prepulse genotype × sex, *p *= 0.0022, *F*_(1,47)_ = 10.54). With a 75 dB prepulse, *Cntnap2* KO males showed a greater change in ES50 than WT males, but *Cntnap2* KO females did not differ from WT females (males, *p *= 0.0039; females, *p *= 0.7418; Fig. 6*C*). With an 85 dB prepulse, *Cntnap2* KO males showed again a greater change in ES50 than WT males; however, *Cntnap2* KO females showed a reduced change in ES50 compared with WT females (males, *p *= 0.0186; females, *p *= 0.0333).

For the change in saturation, there was a main effect of sex with a 75 dB prepulse and an interaction between genotype and sex with an 85 dB prepulse (75 dB prepulse sex, *p *= 0.0001, *F*_(1,47)_ = 17.04; 85 dB prepulse genotype × sex, *p *= 0.0284, *F*_(1,47)_ = 5.116). The change in saturation did not differ between male and female *Cntnap2* KO rats and their WT counterparts with a 75 dB prepulse, and there was only a trend for a lesser change in saturation in *Cntnap2* KO females with an 85 dB prepulse (males 75 dB prepulse, *p *= 0.9945; females 75 dB prepulse, *p *= 0.1150; males 85 dB prepulse, *p *= 0.4956; females 85 dB prepulse, *p *= 0.0577; Fig. 6*D*).

Additionally, we examined the change in slope from the baseline startle curve upon the addition of a prepulse. With both a 75 and 85 dB prepulse, there was no main effect of sex or interaction effect between genotype and sex on the change in slope (75 dB prepulse sex, *p *= 0.0642, *F*_(1,47)_ = 3.593; 75 dB prepulse genotype × sex, *p *= 0.8623, *F*_(1,47)_ = 0.0304; 85 dB prepulse sex, *p *= 0.6879, *F*_(1,47)_ = 0.1634; 85 dB prepulse genotype × sex, *p *= 0.5399, *F*_(1,47)_ = 0.3813). Consequently, sex was collapsed to examine the effect of genotype. *Cntnap2* KO rats had a greater change in slope than WT rats with a 75 dB prepulse (*p *= 0.0438; data not shown). With an 85 dB prepulse, the change in slope did not differ between *Cntnap2* WT and KO rats (*p *= 0.6419). Generally, this again indicates that sound scaling is not uniformly affected in *Cntnap2* KO rats, at least with a 75 dB prepulse.

In summary, sound scaling by a prepulse appeared to be not only intact but even more pronounced in *Cntnap2* KO rats at lower startle stimulus intensities. This was evident in a more pronounced shift in threshold and ES50 when a prepulse was preceding the startle stimulus. However, with an 85 dB prepulse, female *Cntnap2* KO rats showed a reduced change in ES50 compared with WT rats, and there was also a trend to reduced change in saturation. This suggests that there are mild sound scaling deficits in *Cntnap2* KO rats that are restricted to higher startle stimulus intensities, whereas at low startle stimulus intensities, sound scaling is intact or even increased.

### Prepulse inhibition deficits are dependent on startle stimulus intensities

**Figure 7.**
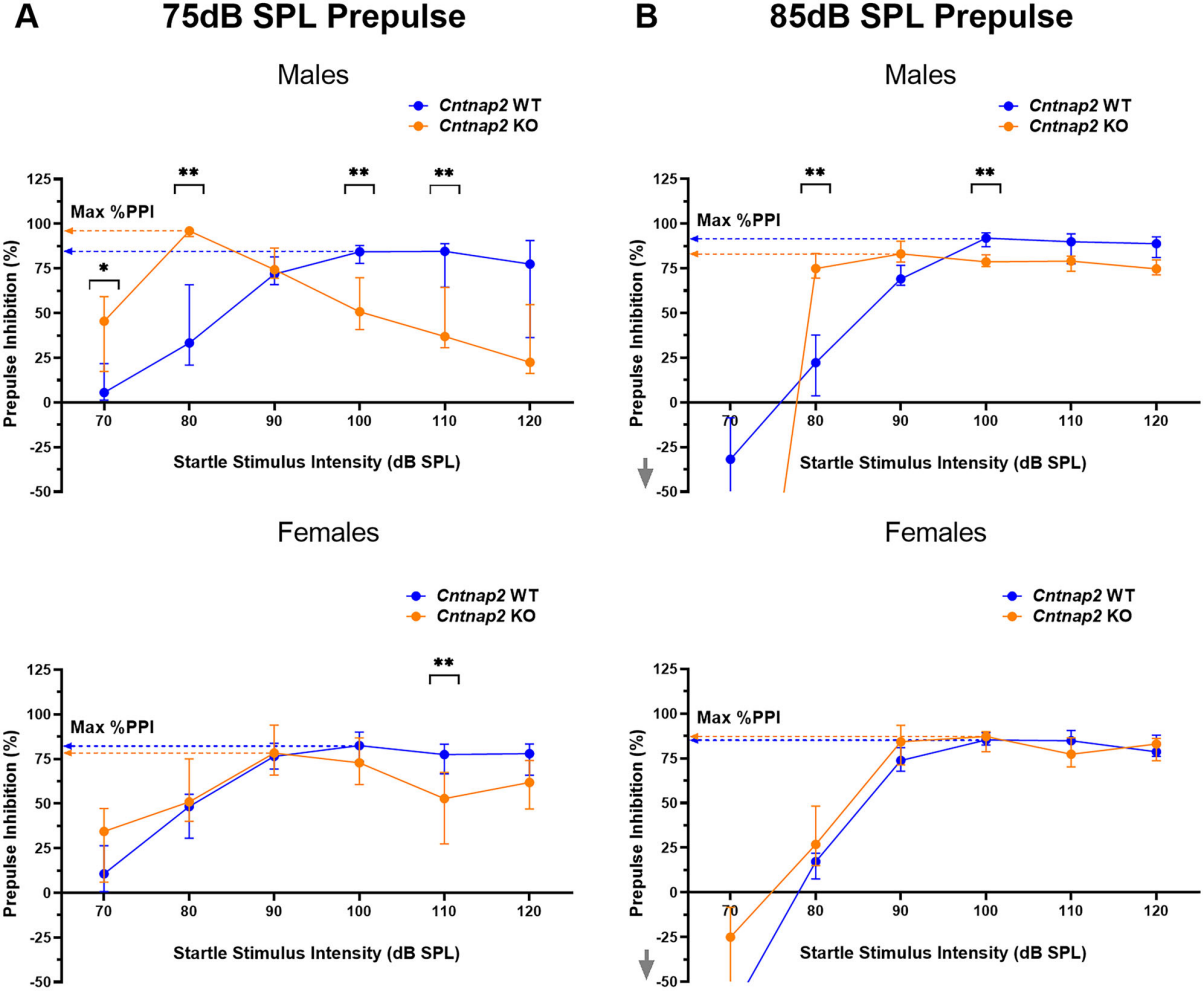
*Cntnap2* KO rats do not have a general PPI deficit. PPI impairment is dependent on prepulse and startle stimulus intensity. *Cntnap2* WT rats are represented in blue and *Cntnap2* KO rats in orange. All graphs show group medians from the 40 repetitions per startle stimulus SPL with error bars representing IQR. ***A***, %PPI across startle stimulus intensity levels (70, 80, 90, 100, 110, and 120 dB) with the 75 dB prepulse. Arrows point to the maximum %PPI values for each group irrespective of startle stimulus intensity. Male *Cntnap2* KO rats showed a PPI deficit at 100 and 110 dB startle stimulus but showed very high levels of PPI at a startle stimulus amplitude of 80 dB. Female *Cntnap2* KO rats showed a PPI deficit at 110 dB startle stimulus. ***B***, %PPI analysis across startle stimulus intensity levels (70, 80, 90, 100, 110, and 120 dB) with the 85 dB prepulse. Arrows point to the maximum %PPI values for each group irrespective of startle stimulus intensity. Male *Cntnap2* KO rats showed a PPI deficit at 100 dB startle stimulus but again, showed a very high level of PPI at 80 dB startle stimulus amplitude. Female *Cntnap2* KO rats did not exhibit PPI deficits at any of the startle stimulus intensities. *p < 0.05, **p < 0.01, ***p < 0.0001, no asterisk or ns indicates nonsignificance of the comparison.

The results of the sound scaling analysis were surprising, as they showed increased sound scaling in *Cntnap2* KO rats that would be expected to result in overall increased PPI at lower startle intensities. Therefore, we used the classical %PPI assessment across the entire range of startle stimulus levels (70, 80, 90, 100, 110, and 120 dB) with a 75 and 85 dB prepulse (Fig. 7*A*,*B*). With the 75 dB prepulse, a significant interaction effect was found between sex and startle stimulus level, as well as between sex, genotype, and startle stimulus level (sex × startle stimulus, *p *< 0.0001, *F*_(5,235)_ = 13.70; sex × genotype × startle stimulus, *p *< 0.0001, *F*_(5,235)_ = 6.230). Similar interaction effects in addition to a main effect of sex and an interaction effect between sex and genotype were found with an 85 dB prepulse (sex, *p *< 0.0001, *F*_(1,47)_ = 141.2; sex × genotype, *p *< 0.0001, *F*_(1,47)_ = 303.9; sex × startle stimulus, *p *< 0.0001, *F*_(5,235)_ = 64.59; sex × genotype × startle stimulus, *p* < 0.0001, *F*_(5,235)_ = 73.81). Data was therefore analyzed separately for males and females.

With a 75 dB prepulse, there was a significant effect of startle stimulus and an interaction effect between genotype and startle stimulus in both males and females (male startle stimulus, *p *< 0.0001, *F*_(5,110)_ = 30.20; female startle stimulus, *p *< 0.0001, *F*_(5,125)_ = 32.03; male genotype × startle stimulus, *p *< 0.0001, *F*_(5,110)_ = 20.93; female genotype × startle stimulus, *p *< 0.0001, *F*_(5,125)_ = 6.545). With an 85 dB prepulse, similar significant effects of startle stimulus and interaction effects between genotype and startle stimulus were found in males and females in addition to a significant effect of genotype (male genotype, *p *< 0.0001, *F*_(1,22)_ = 60.38; female genotype, *p *= 0.0013, *F*_(1,25)_ = 13.12; male startle stimulus, *p *< 0.0001, *F*_(5,110)_ = 15.53; female startle stimulus, *p *< 0.0001, *F*_(5,125)_ = 73.82; male genotype × startle stimulus, *p *< 0.0001, *F*_(5,110)_ = 18.26; female genotype × startle stimulus, *p *= 0.0033, *F*_(5,125)_ = 3.764). To determine at which startle stimulus intensities *Cntnap2* KO animals show differences in PPI, post hoc testing was performed within sound levels. Surprisingly, with a 75 dB prepulse and low startle stimulus intensity of 80 dB, *Cntnap2* KO males actually had higher %PPI compared with WT males (*p *= 0.0003; Fig. 7*A*), whereas at higher startle stimulus intensities of 100 and 110 dB, they showed lower %PPI than WT animals (100 dB *p *= 0.0005; 110 dB *p *= 0.0059). *Cntnap2* KO females also showed a lower %PPI than WT females at the 110 dB startle stimulus but did not differ at any of the other startle stimulus intensities (*p *= 0.0089). With the 85 dB prepulse and 80 dB low startle stimulus, *Cntnap2* KO males again showed a higher %PPI than WT males (*p *= 0.0001; Fig. 7*B*), whereas at the higher startle stimulus intensity of 100 dB, *Cntnap2* KO males had a lower %PPI than WT males (*p *= 0.0008). For females, there were no significant post hoc comparisons within startle stimulus levels for an 85 dB prepulse, indicating no differences between genotypes with this stronger prepulse. In summary, *Cntnap2* KO rats showed remarkably high %PPI at low startle stimulus intensities, where WT animals do not even startle, whereas they showed reduced %PPI at high startle stimulus amplitudes commonly used for PPI testing, in accordance with previous reports of PPI disruptions. Both effects were much more pronounced in male *Cntnap2* KO rats than in females.

Lastly, we determined whether there were differences between genotypes in the maximum %PPI, regardless of stimulus intensity. For each animal, the highest %PPI median from all the sound levels was determined. With a 75 dB prepulse, there was a significant main effect of genotype and sex on the maximum %PPI (genotype, *p *= 0.0237, *F*_(1,47)_ = 5.468; sex, *p *= 0.0010, *F*_(1,47)_ = 12.36). With an 85 dB prepulse, there was an interaction effect between genotype and sex on the maximum %PPI (genotype × sex, *p *= 0.0335, *F*_(1,47)_ = 4.799). *Cntnap2* KO males had a higher maximum %PPI than WT males with a 75 dB prepulse (*p *= 0.0065). However, with an 85 dB prepulse, *Cntnap2* KO males were trending toward having a lower maximum %PPI than WT males (*p *= 0.0840). The maximum %PPI of *Cntnap2* KO females did not differ from WT females for either prepulse intensity (75 dB prepulse, *p *= 0.9059; 85 dB prepulse, *p *= 0.6556).

Overall, this demonstrates that *Cntnap2* KO rats can show remarkably high %PPI, clearly indicating that they do not have a general deficit in sensorimotor gating. Instead, male *Cntnap2* KO rats exhibit even increased maximum PPI capacity compared with WT rats at low startle stimulus amplitudes and with a 75 dB prepulse. Therefore, the PPI deficit consistently reported in *Cntnap2* rats, and potentially in many other animal models, might not reflect a general PPI deficit but a shift in optimal parameters to measure PPI.

## Discussion

The present study utilized a new comprehensive method for assessing startle and PPI, aiming to gain deeper insights into sensory processing deficits in the *Cntnap2* KO rat model of ASD. Our findings revealed that sex, prepulse intensity, and startle stimulus intensity significantly impact startle and PPI. Notably, our results suggest that the detection of a PPI deficit in an animal model is dependent on the specific testing conditions employed. This observation likely contributes to the variability observed in PPI testing results across rodent models of ASD and other neurodevelopmental conditions.

### Baseline startle is differentially affected in male and female *Cntnap2* KO rats

Previous work by El-Cheikh Mohamad et al. (2023) reported that the greater baseline startle response of *Cntnap2* KO rats resulted from both an increase in response magnitude (startle scaling) and a left shift of the I/O function to higher sound sensitivity (sound scaling). However, in the present study with our comprehensive examination of sex effects, we found that the combination of both effects only existed in males. Increased baseline startle in *Cntnap2* KO females is associated with higher startle magnitudes, but not with a left shift of the startle I/O function. Considering that startle and sound scaling pertain to the motor and sensory components of the startle response, respectively, it appears that different mechanisms contribute to the heightened startle in male and female *Cntnap2* KO rats.

Indeed, in vivo extracellular electrophysiological recordings of sound-evoked activity in the PnC of *Cntnap2* KO rats unveiled sex-specific effects that could potentially explain our findings (Zheng et al., 2023). Female *Cntnap2* KO compared with WT rats had significantly higher firing rates in response to acoustic startle stimuli, but the differences between male *Cntnap2* KO and WT rats were minimal. In contrast, it was shown that in males, there exists an increased recruitment of startle-mediating neurons in response to sound (Zheng et al., 2024). It is intriguing to speculate that PnC hyperactivity in females contributes to their increased startle response through startle scaling alone, whereas in *Cntnap2* KO males there is also an increased recruitment of startle mediating neurons, which might increase the sensitivity to sound related to the left shift of the startle I/O function.

Previous findings can dissuade some other potential mechanisms causing increased sound sensitivity in *Cntnap2* KO males. First, the left shift of the startle I/O function in males is not likely related to differences in any structures upstream of the PnC, like the cochlea or auditory nerve. Both areas have been found to show normal neural activity in response to sound as assessed by comparing auditory brainstem responses between genotypes (Scott et al., 2018, 2022; Zheng et al., 2023). Furthermore, sound scaling is likely not a result of differential neurotransmitter levels. Although *Cntnap2* KO rats have elevated levels of excitatory and inhibitory neurotransmitters including glutamate at the PnC, this does not differ between sexes (Möhrle et al., 2021). However, there may be sex differences in the number of PnC giant neurons, or the number of excitatory synapses and/or neurotransmitter receptors on PnC giant neurons, ultimately affecting the recruitment of the neurons in the startle pathway.

### PPI of *Cntnap2* KO rats shows intact startle and sound scaling

Our novel approach to PPI allowed us to differentiate between startle and sound scaling components. El-Cheikh Mohamad et al. (2023) reported that *Cntnap2* KO rats had intact startle scaling but impaired sound scaling. Here, we again showed intact startle scaling in *Cntnap2* KO rats. Since startle scaling is thought to relate to motor output and the primary startle pathway, this consistent finding suggests that these are not factors contributing to impaired PPI in *Cntnap2* KO rats. Instead, it is likely that the mechanisms underlying sound scaling are disrupted, causing the observed PPI deficit. Interestingly, our findings suggest that deficits in sound scaling are relatively mild and emerge specifically at higher startle stimulus intensities, whereas we observed intact or even improved sound scaling at the lower end and middle of the startle stimulus range for *Cntnap2* KO rats as evidenced through threshold and ES50 parameters, respectively. Moreover, deficits in sound scaling were only found in female *Cntnap2* KO rats at the middle range of the startle stimulus intensities. We expected these deficits to persist also at the higher range of the startle stimulus intensities, indicated by our measurement of saturation, but there were no statistical differences between genotypes. We still have reason to speculate that a deficit here exists for *Cntnap2* KO rats considering that *Cntnap2* KO rats, unlike their WT counterparts, exhibited no change in the saturation point from baseline with the addition of prepulse (El-Cheikh Mohamad et al., 2023). In the current study, it appears that this was trending toward significance for *Cntnap2* KO females.

### *Cntnap2* KO rats show PPI but require lower startle stimulus intensities

For the first time, we demonstrated that at lower startle stimulus intensities, *Cntnap2* KO rats can exhibit PPI to the same extent as, or better than, WT animals. Therefore, *Cntnap2* KO rats do not have a general sensorimotor gating deficit. PPI deficits only appear at the higher startle stimulus intensities that are typically used during standard PPI testing like 100 and 110 dB. This suggests that the neural mechanisms underlying PPI are functional in principle, but not at startle stimulus intensities typically used to assess PPI.

Based on this finding, impaired PPI in the *Cntnap2* KO rat is likely related to the detection and/or processing of different startle stimulus intensities. The impact of startle stimulus intensity on PPI in humans and mice has been previously investigated by Csomor et al. (2006). They found that the %PPI typically decreases with increasing startle stimulus intensity. However, in populations with an undetectable startle response at the lower end of the startle stimulus intensity range, this relationship trends toward an inverted U shape in which the middle startle stimulus intensities have the highest %PPI. Our %PPI findings for all animal groups revealed this inverted U shape. However, it appeared that the peak in this curve was shifted leftward for the *Cntnap2* KO compared with WT rats, particularly in males. Consequently, higher startle stimulus intensities are typically used in standard PPI testing like 100 and 110 dB, since this is where the maximum %PPI would be found in WT rats. The maximum %PPI in *Cntnap2* KO rats, however, occurs at lower startle stimulus intensities like 80 dB. Since WT rats do typically not even show a startle response at these sound levels, these startle stimulus intensities are normally not included in PPI testing. This emphasizes the need to consider an animal's reactivity profile when determining the startle stimulus intensity range to evaluate PPI. Animal models that exhibit high startle reactivity like the *Cntnap2* KO rat require PPI testing conditions with lower startle stimulus intensities. It would be interesting to analyze the startle and PPI of both genotypes by comparing values of stimulus intensities relative to the respective startle threshold. This would potentially compensate for the left shift of the startle I/O function, but not for the huge difference in maximum startle.

Our results show that *Cntnap2* KO rats do not have a general deficit in PPI—so why do they show impaired PPI at higher startle stimulus intensities? A likely explanation is that at higher startle stimulus intensities, the mechanisms mediating PPI become overwhelmed by the strong signaling in the primary startle pathway, i.e., the inhibition of the startle pathway by the midbrain circuits mediating PPI is not strong enough to effectively inhibit startle. This may arise due to the imbalance of excitatory and inhibitory neurotransmission within the circuits modulating startle and mediating PPI. In fact, it has been shown that *Cntnap2* KO rats have higher levels of glutamate, GABA, and glutamine, the precursor for both glutamate and GABA, in the PnC when compared with WT rats (Möhrle et al., 2021). This suggests that the typical excitatory/inhibitory homeostasis at the PnC is disturbed through dysregulation of the reuptake and/or synthetization cycles of these neurotransmitters (Tebartz van Elst et al., 2014). This could lead to generally higher excitability within the PnC and therefore higher startle. High startle stimulus intensities would trigger an excessive release of glutamate at synapses in the PnC which would be difficult for the inhibitory mechanisms initiated by PPI to counter. Alternatively, the inhibition of the PnC by midbrain circuits mediating PPI could be impaired. It is generally assumed that the attenuation of the startle response through a prepulse is mainly due to inhibitory activity in PPTg neurons projecting to the PnC (Koch, 1999). In vivo electrophysiological recordings in the PPTg and the PnC have shown that the extent of sound-evoked PPTg inhibition on the PnC is not affected in *Cntnap2* KO rats (Zheng et al., 2023). Therefore, it is more likely that impaired inhibition of the PnC is due to a general imbalance of neurotransmitters in the PnC and/or to changes in tonic PnC modulation through projections from secondary brain regions such as the inferior colliculus, superior colliculus, or auditory cortex (Gómez-Nieto et al., 2020).

### Considerations of high response variability in *Cntnap2* KO rats

An important consideration in this study is the high variability in the responses of *Cntnap2* KO rats for both baseline startle and PPI. This high variability observed both intra- and interindividually is common across several behaviors in *Cntnap2* KO rats and other animal models of ASD (Kazdoba et al., 2014; Ergaz et al., 2016; Möhrle et al., 2021; El-Cheikh Mohamad et al., 2023). Given that similar variability is also observed in autistic children, the increased variability in *Cntnap2* KO rats appears to be a phenotype in itself (Vivanti et al., 2014; David et al., 2016; Uljarević et al., 2017). The heightened variability in the startle responses of *Cntnap2* KO rats may interfere with the fit to the applied sigmoidal function. This variability leads to both very small and very large baseline startle responses, impacting the symmetric sigmoid form of the input/output (I/O) response. Although we tried to mitigate this by using a high repetition number for each stimulus, the high variability persisted. Our measure of fitting quality (Sy.x) for the absolute startle and PPI curves was consistently higher for the *Cntnap2* KO rats than for the WT rats. However, this was resolved for the scaled response curves used for the sound scaling parameters where the Sy.x was consistent between *Cntnap2* KO and WT rats.

Overall, the present study demonstrates that startle and PPI deficits in the *Cntnap2* KO rat model for ASD depend on the testing parameters employed. By introducing a new method for assessing startle and PPI, we showed that the *Cntnap2* KO rat does express intact PPI, and in some cases to a greater extent than WT animals, but lower startle stimulus intensities are required. This finding encourages the careful consideration of testing conditions when assessing PPI in not only animal models of ASD and other neurodevelopmental conditions but also extends its relevance to applications in human populations.

## Synthesis

Reviewing Editor: Julie Bakker, University of Liege Decisions are customarily a result of the Reviewing Editor and the peer reviewers coming together and discussing their recommendations until a consensus is reached. When revisions are invited, a fact-based synthesis statement explaining their decision and outlining what is needed to prepare a revision will be listed below. The following reviewer(s) agreed to reveal their identity: NONE.

Synthesis: some minor comments remain, please address these!

General comments:

The ms. has been thoroughly revised according to the suggestions by the reviewers. Several points were clarified as suggested.

The authors provided now more details regarding the separated analysis of 'startle scaling' and 'sound scaling'. Separating this by using normalized startle response curves only for the second part of the analysis still seems to me problematic. The KO-induced increase in startle response magnitude (compared to WT) is small for low startle stimulus SPL and increases nonlinearly to a factor of around 4 at high SPL, resulting in the asymmetrical growth functions.

1. The data now provided about the standard error of regression shows that there is indeed a problem with fitting sigmoid functions to the data from KO animals (especially male). To my opinion this is clearly a non-symmetric growth function (see baseline functions from KO animals in Fig. 5A) that should be described by a generalized logistic function (often named 5-parameter sigmoid fitting). The much higher Sy.x-values for KO data support this notion (from captions to Fig. 3: Goodness of fit Sy.x: male Cntnap2 WT = 449.7, male Cntnap2 KO = 2336)

2. This has a strong effect on the derived 'threshold' parameter from the curves. I strongly suggest that the authors use their tools for post-hoc analysis in two ways to provide further insight on the change in threshold:

a) for non-normalized data: which is the lowest SPL providing (in post-hoc analysis) significantly higher startle responses compared to the 'baseline data' at 70 dB SPL?

b) same for normalized data.

Specific comments:

Introduction:

Page 2/line 63f: The statement "Startle and sound scaling are thought to relate to the ..." is mixed up. Sound scaling is related to sensory processing.

Methods:

p. 3 / l. 125 X-values for the fitting are not "concentration". Adjust description to this specific case.

## Author Response

Reviewers Comments (2) - Responses 1. The data now provided about the standard error of regression shows that there is indeed a problem with fitting sigmoid functions to the data from KO animals (especially male). To my opinion this is clearly a non-symmetric growth function (see baseline functions from KO animals in Fig. 5A) that should be described by a generalized logistic function (often named 5-parameter sigmoid fitting). The much higher Sy.x-values for KO data support this notion (from captions to Fig. 3: Goodness of fit Sy.x: male Cntnap2 WT = 449.7, male Cntnap2 KO = 2336) We agree that the KO animals, particularly the males, exhibit asymmetrical curves. We appreciate the suggestion of fitting the data to a five-parameter logistic equation (5PL) rather than the four-parameter logistic equation (4PL), and we were excited to try a 5PL fitting procedure, despite the fact that studies in the last 40 years have always used a 4PL fitting procedure. We tried this with our data following instructions provided by Prism (https://www.graphpad.com/guides/prism/latest/curve-fitting/reg_asymmetric_dose_response_ec_2.htm). We examined the Sy.x values for the 4PL and 5PL for each animal group and they do not differ, in fact, the 5PL is often worse (see below). As mentioned in our manuscript discussion section, startle and PPI of Cntnap2 KO rats are highly variable, a phenotype we see also in autistic children. We therefore attribute the differences in Sy.x values between the WT and KO rats observed with the 4PL fit mainly to differences in variation. Furthermore, as described in the Prism manual, 5PL fits require a large amount of high-quality data, so after exploring 5PL fitting, we are not confident that a 5PL fit is a better way to analyse the data.

To better understand why the Sy.x values were so similar between 4PL and 5PL, we scrutinized the 5PL equation where variable S is introduced as the unitless symmetry parameter:

When S = 1, the curve is symmetrical and identical to 4PL. For most of our animals, both WT and KO, S was an extremely large value, many in the trillions. High noise or variability in the data can obscure the asymmetry, leading the model to have a high S value. When this occurs, the potential asymmetry is essentially ignored, and the equation resembles a 4PL fit more closely. This is because when S is high, the term 2(1/S)-1 approaches 1 and 〖〖((ES50/X)〗^Hillslope)〗^S becomes dominant.

We recognize that one way to potentially address this issue is to have a greater range of startle stimuli. Adding higher startle stimuli might make the data stable enough to provide more accurate S values and a good fit for 5PL. However, repeated testing with more than 120 dB has been shown to be damaging to the hearing of the animals. We made the conservative choice of using a maximum startle level of 120 dB.

In summary, after exploring 5PL fitting, we conclude that 4PL remains the best fit for this data.

2. This has a strong effect on the derived 'threshold' parameter from the curves. I strongly suggest that the authors use their tools for post-hoc analysis in two ways to provide further insight on the change in threshold: a) for non-normalized data: which is the lowest SPL providing (in post-hoc analysis) significantly higher startle responses compared to the 'baseline data' at 70 dB SPL? b) same for normalized data.

We did this analysis as suggested and summarized the findings below - the table displays the lowest startle stimulus level with a significantly higher startle response than at 70 dB for the baseline and prepulse curves.

We do not think adding this analysis will improve the manuscript. The 10 dB increases in sound level do not give sufficient resolution to detect changes in threshold reliably using this type of analysis.

Minor edits:

1. Page 2/line 63f: The statement "Startle and sound scaling are thought to relate to the ..." is mixed up. Sound scaling is related to sensory processing.

Corrected.

2. p. 3 / l. 125 X-values for the fitting are not "concentration". Adjust description to this specific case.

On Prism, the name of the model used is "Sigmoidal, 4PL, X is concentration". We think this should remain in the manuscript to facilitate the replication of this method.
